# JITC launches a new section: commentary and editorials

**DOI:** 10.1186/s40425-015-0073-1

**Published:** 2015-06-24

**Authors:** Christian M. Capitini, Pedro Romero

**Affiliations:** Department of Pediatrics and Carbone Comprehensive Cancer Center, University of Wisconsin-Madison, Madison, WI USA; Translational Tumor Immunology Group, Ludwig Cancer Research Center, University of Lausanne, Lausanne, Switzerland

## Editorial

### Background

We are pleased to announce the launch of a new section in the *Journal for ImmunoTherapy of Cancer* (JITC), the Commentary/Editorials section edited by Christian Capitini, MD of the University of Wisconsin. With all of the changes occurring in the field of immunotherapy, we felt having a section that features expert analysis and insight into the latest developments would be useful to our readers.

So far in 2015, we have seen mounting excitement around recent Food and Drug Administration (FDA) approvals for new immunotherapeutics for cancer. In March of this year, two monoclonal antibodies (mAbs) were approved. The first approval was for the PD-1 pathway inhibitor nivolumab, which was approved for metastatic non-small cell lung cancer (NSCLC). Nivolumab was reviewed under the FDA’s priority review program. The second approval involved the anti-GD2 therapy dinutuximab, which was approved for children with high-risk neuroblastoma. Dinutuximab was granted priority review and orphan product designation, and became the second drug granted a rare pediatric disease priority review voucher. It is only the third drug in history to receive initial approval for the treatment of a pediatric cancer.

In addition, we have seen promising reports from early phase immunotherapy studies presented at the American Association for Cancer Research (AACR) earlier this year. In the ever-expanding field of mAbs for cancer, the first phase III trial comparing the PD-1 inhibitor pembrolizumab versus the CTLA4 inhibitor ipilimumab as first-line therapy demonstrated that pembrolizumab was superior in patients with metastatic melanoma according to all of the study endpoints [1]. Moreover, recent analyses of whole exome sequencing of NSCLC tumors treated with pembrolizumab shows that higher mutational burden, a molecular smoking signature, higher neoantigen burden and DNA repair pathway mutations improves efficacy [2]. Perhaps these results, combined with data showing that presence of CD8^+^ T cells expressing PD-1 at the tumor margin is associated with tumor regression [3], will improve access to anti-PD-1 therapies for these patients, especially in Europe where anti-PD-1 therapy is not currently available as a first-line therapy. In a phase I trial in patients with metastatic NSCLC, pembrolizumab was found to be safe. Remarkably in a cohort of patients with PD-L1^+^ tumors, the response rate was 45 % [4]. Beyond melanoma and NSCLC, in a separate phase Ib trial, pembrolizumab was reported to be safe for patients with malignant pleural mesothelioma. Some clinical responses were reported.

MPDL3280A is a PD-L1 inhibitor that was previously shown to induce response in metastatic bladder cancer [5]. In a phase I clinical trial, MPDL3280A was reported to be safe and showed some durable clinical activity in patients with metastatic triple-negative breast cancer. In a phase II, double-blind trial, the combination of ipilimumab and nivolumab was reported to show better objective clinical responses and progression-free survival than ipilimumab alone [6] And in a phase I clinical trial, the anti-CD40 monoclonal antibody CP-870,893 was combined safely with the CTLA4 inhibitor tremelimumab in patients with metastatic melanoma, with non-overlapping side effects reported as well as clinical responses. In a phase I/IIa trial, a monoclonal T cell Receptor anti-CD3 scFv Fusion Protein IMCgp100 showed durable responses in melanoma. In the field of adoptive cell therapy, chimeric antigen receptor (CAR)-modified T cells against mesothelin were well tolerated across patients with multiple tumor types. Importantly, no off-tumor, on-target toxicity was reported.

## Conclusion

With the rapid expansion of mAb technologies such as antibody-drug conjugates, immunotoxins, immunocytokines, radiolabeled-mAb, and bispecific antibodies, and cell-based therapies like CAR-modified T cells, tumor-infiltrating lymphocytes (TILs), T cell receptor (TCR)-transduced T cells, natural killer cells, and dendritic cell vaccines, there will be lots to discuss in the coming months (and years) ahead. We at JITC share your interest and enthusiasm, and are prepared to keep you well informed of the progress made in the immunotherapy of cancer.
